# Society for Healthcare Epidemiology of America supports environmental stewardship and sustainability while protecting patients and healthcare personnel position statement of the SHEA Board

**DOI:** 10.1017/ash.2025.10262

**Published:** 2025-12-26

**Authors:** David J. Weber, Priya Nori, Rajalakshmi Ananthanarayanan, Carolee Estelle, Jesse T. Jacob, Jennie H. Kwon, Lisa L. Maragakis, Rekha Murthy, Ann-Christine Nyquist, Catherine Passaretti, Lisa Saiman, Erica S. Shenoy, Julia E. Szymczak, Thomas R. Talbot

**Affiliations:** 1 University of North Carolina at Chapel Hill, Chapel Hill, NC, USA; 2 Montefiore Health System, Albert Einstein College of Medicine, Bronx, NY, USA; 3 KIMSHEALTH, Kerala, India; 4 University of Texas Southwestern & Parkland Health, Dallas, TX, USA; 5 Emory University, Atlanta, GA, USA; 6 Northwestern University Feinberg School of Medicine, Chicago, IL, USA; 7 Johns Hopkins University School of Medicine, Baltimore, MD, USA; 8 Cedars-Sinai Hospital, Los Angeles, CA, USA; 9 Children’s Hospital Colorado, University of Colorado Medicine, Aurora, CO, USA; 10 Advocate Health, Charlotte, NC, USA; 11 Columbia University Irving Medical Center, NewYork-Presbyterian Hospital, New York, NY, USA; 12 Mass General Brigham, Massachusetts General Hospital and Harvard Medical School, Boston, MA, USA; 13 University of Utah School of Medicine, Salt Lake City, UT, USA; 14 Vanderbilt University Medical Center, Nashville, TN, USA

The leading cause of climate change and global warming is the increase in greenhouse gas emissions.^1^ The health impacts of climate change and extreme weather events include temperature-associated illnesses and deaths, air pollution-associated chronic respiratory illness, water-and foodborne infections, and vector-borne and zoonotic infections.^2^

There is a clear link between climate change and SHEA’s mission-critical concerns, such as healthcare-associated infections, antimicrobial resistance (AMR), and the spread of *Candidozyma auris* within healthcare settings.^3^ Moreover, climate change increases the frequency and severity of bacterial, fungal, and vector-borne diseases including gastroenteritis, skin and soft tissue infections, and respiratory illnesses.^4^ Broader infectious disease and public health threats resulting from climate change include the northward spread of *Vibrio vulnificus* along the Atlantic coastline and a global expansion of mosquito vectors with local transmission of malaria and dengue.^5,6^

Therefore, as healthcare epidemiologists, infection preventionists and antibiotic stewards concerned with public health and providing safe healthcare to all, it is incumbent on us to reduce the driving factors of climate change within our domains and mitigate adverse health impacts.

## Antibiotic and diagnostic stewardship

There is a clear synergy between climate change, AMR, and their magnified impacts on the world’s most vulnerable populations.^7^ Achieving target reductions for global warming and AMR are interlinked goals of the United Nations global agenda.

Antimicrobial stewardship (AS) is an extension of frontline patient care and directly affects individual and population-level outcomes. Interventions to reduce excess antibiotic days of therapy, including shorter antibiotic courses, earlier de-escalation of broad-spectrum agents or intravenous (IV) to oral (PO) switch, can reduce plastic waste associated with IV administrations and have a positive and direct impact on reducing healthcare-associated carbon emissions.^8^ Moreover, measures to improve diagnostic stewardship by avoiding unnecessary diagnostic testing or leveraging reflexive testing have downstream positive impacts on reducing plastic waste and energy consumption while improving patient outcomes. Therefore, all stewardship programs should implement and scale up proven interventions like de-escalation/cessation, IV to PO switch, and diagnostic stewardship to reduce healthcare utilization and waste and improve patient outcomes. Stewardship programs should also track cost from excess antimicrobial use as quality measures of programmatic success. Nelson et al reported that a switch from frequently dosed meropenem to once daily ertapenem for extended spectrum β-lactamase (ESBL) bloodstream infections resulted in comprehensive cost savings (drug costs, IV tubing, saline, vials, etc.), decreased nursing time, a 66% reduction in waste, and a mean length of stay reduction of >3 days.^9^ To assist stewardship programs with their sustainability efforts, carbon emission reductions can also be tracked. A novel carbon emissions estimation tool has been developed for disposable waste associated with antimicrobial packaging, preparation, and administration in the hospital setting.^10^ Numerous successes of the stewardship community demonstrate that we can achieve optimal patient outcomes from infectious diseases while also implementing safe and sustainable practices (Table 1).

**Table 1.** Noteworthy healthcare sustainability-focused interventions and studies

Intervention	Outcome/details
**Antimicrobial stewardship**	
Chakravorty et al quantified emissions from excess days of IV antibiotics in patients eligible for PO antibiotics (per institutional IV to PO switch protocol). ^17^	An estimated 9,694 IV days of therapy could have been saved, amounting to 2,049 kg of solid waste, or 0.353 metric tons of CO_2_ equivalents, 904 miles driven, 40 gallons of gasoline consumed, or 389 pounds of coal burned.
Spivak et al measured greenhouse gas emissions from unnecessary outpatient antibiotic prescriptions.^8^	13,580,000 estimated unnecessary prescriptions in the United States approximating 993,906 miles driven in a gas-powered car
Hojat et al led a multi-society effort to develop a calculator to compare carbon emissions of various IV or PO antimicrobial regimens.^18^	Antibiotic Waste Calculator
In 2023, the “Sustainabil-ID” interest group of pediatric and adult infectious diseases providers was established to promote sustainability interventions in ID. ^18,19^	Member Spotlight: Preeti Jaggi and Shreya Doshi—Pediatric Infectious Diseases Society
**Infection prevention and control**	
Lalakea et al implemented an educational intervention on hand hygiene, appropriate glove use, and environmental impacts to reduce non-sterile glove overuse in surgical specialty outpatient clinics.^11^	An average of 4719 gloves saved per month with a total savings of 56,628 gloves, 180.2 kg of waste and $3,003.17 per year, with projected 1472–1767 kg reduction in CO_2_ emissions, equivalent to 3766–4519 miles driven in a gas-powered car.
Pearl et al conducted a SHEA Research Network survey of hospital epidemiologists, IPC directors, and infection preventionists on knowledge, attitudes, and institutional practices related to environmental sustainability and IPC.^20^	Most respondents supported or had in place sustainable measures like donation of gently used or unused medical supplies, pursuit of LEED green buildings certification, water/energy conservation, reusable PPE, and “greener” chemicals for low-level disinfection
Greene et al developed a conflict-based pragmatic approach to reducing single-use plastics in healthcare, which can be applied to AS, and IPC-based interventions, among others.^21^	“No conflict”—immediately implementable “Context-dependent”—requires further exploration “Value conflict”—too high risk/too many safety concerns

Note. ID, infectious disease; IPC, infection prevention and control; IV, intravenous; LEED, leadership in energy and environmental design; PO, orally administered.

## Healthcare epidemiology and infection prevention and control

Disposable gloves are the highest-volume single-use product used in healthcare.^11^ Therefore, Infection Prevention and Control (IPC) programs should continue to recommend their use only when indicated to protect healthcare personnel or patients. For example, the Centers for Disease Control and Prevention (CDC) notes that Occupational Safety and Health Administration “regulations do not require gloves to be worn when administering vaccinations, unless persons administering vaccinations have open lesions on their hands or are likely to come into contact with a patient’s body fluids.”^12^ Exploring conversion from disposable to reusable isolation gowns, when feasible and acceptable, can reduce energy consumption, greenhouse gas emissions, and solid waste generation.^13^ Finally, IPC programs can continue to promote the disposal of healthcare waste in the appropriate receptacle, given that biohazardous waste generates more greenhouse gas emissions due to the high energy required for autoclaving or incineration.^14^

However, measures designed to improve healthcare sustainability must be balanced with risks of communicable disease transmission in healthcare settings. Examples of interventions designed to mitigate the effects of climate change or improve sustainability reported as leading to healthcare-associated infections have included use of electronic faucets in an intensive care unit^15^ and the installation of a low-flow recirculated hot water system in a new hospital.^16^ When evaluating competing methods of protecting healthcare personnel or patients, facilities must consider the impact of each alternative. For example, when considering the use of disposable versus reusable endoscopes (ie, bronchoscopes and gastrointestinal endoscopes), consider the following: (1) multiple outbreaks have been reported of multidrug-resistant gram-negative bacilli transmitted by reusable endoscopes despite high-level disinfection that adhered to the manufacturer’s instructions for use, (2) current technology has not validated a method of low-temperature sterilization or high-level disinfection that can guarantee a pathogen-free duodenoscope, and (3) use of a sterile disposable duodenoscope is a proven safe and effective method of endoscopy.

SHEA recommends that IPC and AS programs prioritize sustainability while assessing benefits and potential risks of new products, or new policies and procedures (Table 1). Importantly, IPC and AS experts should examine whether implementation of sustainable strategies could have unintended harms related to the risk of healthcare-associated infections and pathogen transmission within the healthcare setting and develop potential strategies to mitigate such risks.

In summary, the SHEA Board of Trustees commends infection prevention and AS professionals who seek to implement rational, sustainable practices to reduce healthcare’s carbon emissions and improve patient outcomes. SHEA members should be “at the table” for high-level discussions on sustainability-based decisions that may affect our mission of safe healthcare for all. Likewise, SHEA members should promote proven IPC and stewardship interventions which can also reduce costs and excess plastic waste. We can identify opportunities to promote sustainability in our daily work while prioritizing patient and healthcare personnel safety.
